# High Resolution Analysis of Meiotic Chromosome Structure and Behaviour in Barley (*Hordeum vulgare* L.)

**DOI:** 10.1371/journal.pone.0039539

**Published:** 2012-06-25

**Authors:** Dylan Phillips, Candida Nibau, Joanna Wnetrzak, Glyn Jenkins

**Affiliations:** Institute of Biological, Environmental and Rural Sciences (IBERS), Aberystwyth University, Aberystwyth, United Kingdom; National Cancer Institute, United States of America

## Abstract

Reciprocal crossing over and independent assortment of chromosomes during meiosis generate most of the genetic variation in sexually reproducing organisms. In barley, crossovers are confined primarily to distal regions of the chromosomes, which means that a substantial proportion of the genes of this crop rarely, if ever, engage in recombination events. There is potentially much to be gained by redistributing crossovers to more proximal regions, but our ability to achieve this is dependent upon a far better understanding of meiosis in this species. This study explores the meiotic process by describing with unprecedented resolution the early behaviour of chromosomal domains, the progression of synapsis and the structure of the synaptonemal complex (SC). Using a combination of molecular cytogenetics and advanced fluorescence imaging, we show for the first time in this species that non-homologous centromeres are coupled prior to synapsis. We demonstrate that at early meiotic prophase the loading of the SC-associated structural protein ASY1, the cluster of telomeres, and distal synaptic initiation sites occupy the same polarised region of the nucleus. Through the use of advanced 3D image analysis, we show that synapsis is driven predominantly from the telomeres, and that new synaptic initiation sites arise during zygotene. In addition, we identified two different SC configurations through the use of super-resolution 3D structured illumination microscopy (3D-SIM).

## Introduction

Genetic variation in most sexually reproducing organisms is generated during meiosis by reciprocal crossing over between homologous chromosomes, and independent assortment of maternal and paternal chromosomes. Usually, each pair of homologues has at least one crossover to ensure regular bivalent orientation and segregation at the end of the first meiotic division. Crossover interference in many organisms prevents the clustering of crossovers, and effectively caps the numbers of crossovers a bivalent may have. Superimposed on these constraints on crossover frequency and distribution is a phenomenon which confines crossovers to particular chromosome regions in some organisms. Cytological and genetic mapping studies have shown that crossovers are preferentially distributed to distal regions of the chromosomes of many important members of the Poaceae, such as wheat [1], [2], [3], barley [4], [5], [6] maize [7] and ryegrass [8], [9]. Recently, Mayer *et al.* [5] have estimated that 3125 genes of barley map to regions classified as genetic centromeres, and one third (6788) of all genes of the barley genome fall within 10cM of these regions. This corroborates the long-held view that a substantial proportion of the genes of the cereals and grasses are consigned to recombinationally cold regions of the genome, and rarely, if ever, recombines. A restricted pattern of recombination may be beneficial in natural populations, as it would ensure the maintenance of favourable linkage groups, thereby conferring a selective advantage [10]. However distal localisation of chiasmata has the effect of curtailing the potential genetic variation of a species, and has important implications in terms of limiting the scope of map-based cloning approaches and introgression programmes, and the effectiveness of phenotypic selection in advanced breeding programmes. Clearly, there is potentially much to be gained by redistributing crossovers to more interstitial and proximal regions of chromosomes, which is ultimately dependent upon a detailed understanding of the process of meiosis and recombination in these crop species.

Much of our understanding of the genetic control of meiosis has come from genetic, cytological and molecular biological studies of model organisms, such as *Saccharomyces cerevisiae* [11], [12] and *Arabidopsis thaliana* [13], [14], which have given us through translational approaches unprecedented access to meiosis in less tractable organisms, such as wheat [15], [16], [17], rye [18], [19], barley [20] and maize [21], [22]. Much of this work has targeted early events in meiosis which appear to determine the conditions necessary for successful homologue recognition, pairing, synapsis and recombination. At the onset of meiosis in many organisms, centromeres and telomeres are partitioned in the nucleus in a Rabl orientation [23] which is presumed to be a relic of anaphase segregation of chromosomes in the pre-meiotic division. The transition from the Rabl orientation to the clustering of telomeres in a bouquet arrangement, which is defined as a cluster of telomeres that occupies a limited region of the nuclear envelope, occurs during leptotene in many organisms and is thought to be a process which heralds or facilitates homologue recognition [24]. The non-random distribution of centromeres and telomeres at this stage of meiosis has been especially well scrutinised in polyploid wheat as it is implicated in the mechanism of diploidisation in these plants. Twenty one pairs of homologous or non-homologous centromeres formed before meiosis cluster into seven groups of six at the onset of meiosis. These six groups resolve into pairs of homologous centromeres at the same time as the bouquet is formed [25], [26], [27], [28].

The synapsis of homologues is defined by the assembly of synaptonemal complexes (SCs) during zygotene. These tripartite, proteinaceous structures are remarkably well conserved structurally in the animal and plant kingdoms, and provide the framework for recombination events [29], [30], [31]. Until recently, much of our understanding of the progression of SC formation has been gleaned from electron microscopy (EM), which provides the resolution necessary to probe SC substructure. The molecular characterisation of SC components and associated recombination proteins has provided not only important insights into the genetic control of meiosis and crossover formation, but also clarified the relationship between SCs and the recombination process. Furthermore, the availability and effectiveness of antibodies to many SC and recombination proteins have enabled detailed studies of their spatio-temporal expression and the molecular assembly of meiotic prophase chromosomes [12]. Two antibodies to SC structural proteins of *Arabidopsis* (ASY1 and ZYP1) have particular utility in this respect [32], [33], as they bind with great fidelity to orthologous proteins in other species [18], [20], [34], and enable the fluorescence imaging of the molecular assembly of the SC during prophase I. The ASY1 protein itself is not an integral part of the AEs and LEs, and is described as being associated with these components [32], [35], [36]. The precise function of the ASY1 protein is currently unknown although it has been shown in *Arabidopsis* that the ASY1 protein initially binds to chromatin during leptotene prior to AE formation [32]. This observation led to the suggestion that the ASY1 protein acts as the interface between the axis-associated chromatin and the SC.

Barley is a self-fertile, diploid (2n = 2x = 14) monocot of the Poaceae. It has considerable agronomic importance globally, being ranked fifth in world food production (http:/faostat.fao.org/). Despite the importance of this cereal, our understanding of its meiosis is still in its infancy. This study begins the detailed exploration of meiosis in this species by targeting early events, such as homologue recognition and synapsis. We show that the bouquet of barley forms during leptotene and that centromeres associate non-homologously at this time. We demonstrate by monitoring the molecular assembly of the SCs that synapsis is driven from the telomeres, and that additional synaptic initiation sites are added throughout zygotene. We also explore the organisation of the SC by super-resolution 3D structured illumination microscopy (3D-SIM) and identify two different SC configurations.

## Results

### Centromere and telomere behaviour during prophase I

To enable a quantitative 3D analysis of the nuclear behaviour of these chromosome domains at early meiotic prophase, barley meiocytes were embedded in polyacrylamide and hybridised *in situ* with centromere and telomere probes. From a sample of 170 leptotene nuclei, 130 had distinct polarisation of centromere and telomere signals, with clustering of centromere signals (mean 8.1; SD 1.7, range 5–14) and relatively dispersed telomere signals (mean 20.0; SD 6.8, range 6–30). 40 nuclei with a distinct aggregate of telomere signals (mean 8.6; SD 2.9, range 4–20) represent the bouquet stage of meiosis, and had on average 7.5 (SD 1.5, range 5–11) centromere signals.

**Figure 1:**
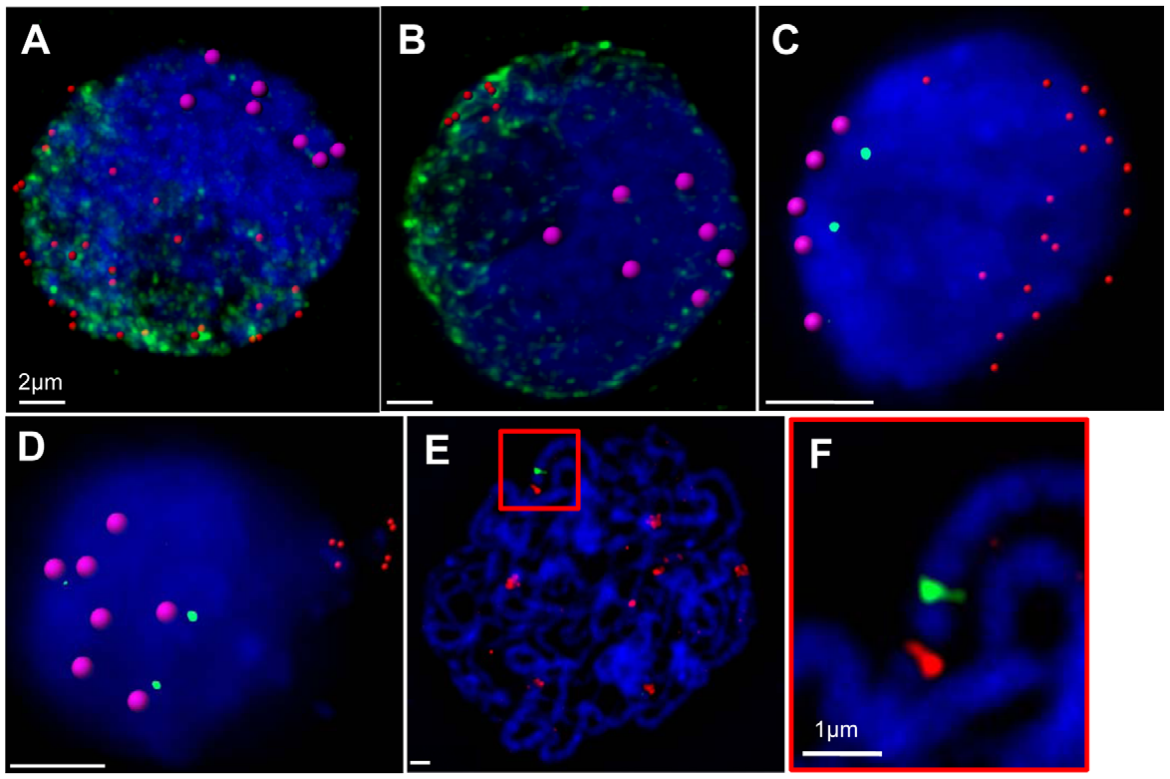
Centromere and telomere behaviour during prophase I. (A) Leptotene nucleus with 28 rendered telomere signals (red), 7 rendered centromere signals (pink) and diffuse, polarised ASY1 signals (green). (B) Leptotene nucleus with a cluster of 7 rendered telomere signals (red), 7 rendered centromere signals (pink) and linearising elements of ASY1 (green) in the same hemisphere as the telomeres. (C) FISH to an embedded leptotene nucleus with a Rabl orientation of 19 rendered telomere signals (red) and 5 rendered centromere signals (pink), and 2 separate single-locus BAC DH053N18 signals (green). (D) FISH to an embedded leptotene nucleus with a bouquet orientation of 7 rendered telomere signals (red) and 7 rendered centromere signals (pink), and 2 separate single-locus BAC DH053N18 signals (green). (E) FISH to a squashed pachytene nucleus showing centromeres (red) and a single BAC signal (green). (F) Enlargement of red box shown in E, showing the close proximity of the BAC (green) to the centromere (red). All images are deconvolved maximum projections of nuclei, and are counterstained with DAPI (blue). (A–B) were imaged by CLSM, and (C–G) by wide-field fluorescence microscopy. (A–D) For ease of counting, the positions of centromeric and telomeric FISH signals have been marked by rendered spears using Imaris, an un-rendered nucleus is shown in Figure S1A.

**Figure 2:**
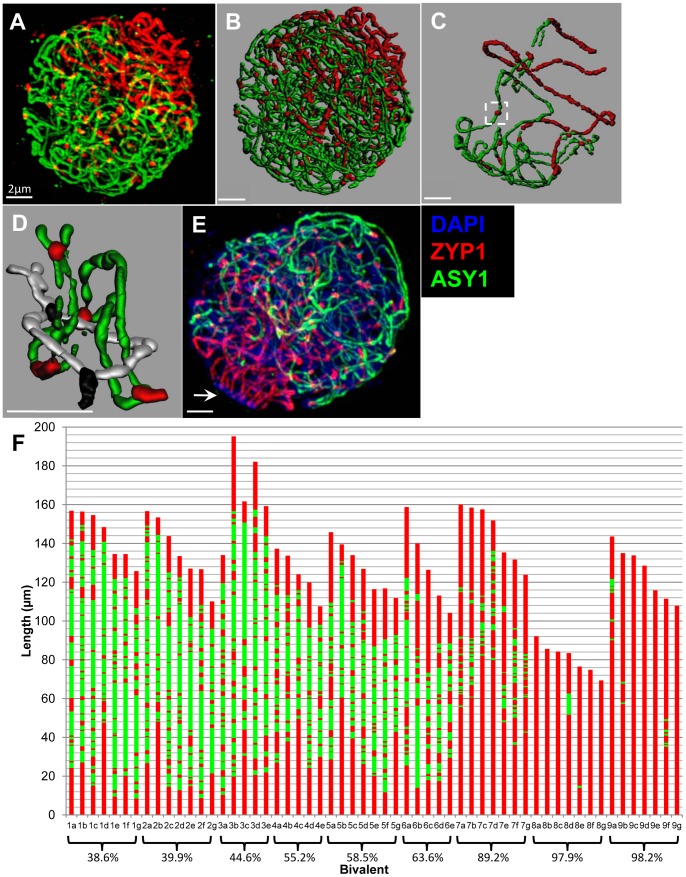
Analysis of synaptic progression during barley prophase I. (A & E) Deconvolved maximum projection of a zygotene nucleus embedded in polyacrylamide and captured using CLSM, showing ASY1 cores (green) and ZYP1 cores (red). (B) Zygotene nucleus shown in Figure 2A processed using Imaris with each of the seven synapsing bivalents isolated, showing generated surfaces for ASY1 (green) and ZYP1 (red). (C) A single bivalent extracted from the reconstruction in Figure 2B, containing an example of a ZYP1 focus present on a single AE (white box). (D) Detail of an interlock isolated from the nucleus shown in Figure 2A. For ease of interpretation, one of the bivalents has been re-coloured to show ASY1 in grey and ZYP1 in black. (E) Zygotene nucleus containing a telomere cluster delimited by darker staining DAPI (blue) and emanating Zyp1 cores identified by the white arrow. (F) Stacked bar graph showing the cumulative lengths of ZYP1 and ASY1 fragments constituting each bivalent isolated from 9 nuclei. Bivalents from the same nucleus are grouped together and ordered by descending length. Bivalent complements are ordered by ascending average percentage synapsis shown below the groups.

In order to correlate centromere and telomere behaviour with the assembly of SC components, and to verify the identity of meiotic nuclei, FISH with centromere and telomere probes was combined with immunolocalisation of ASY1 in embedded meiocytes. Early leptotene nuclei with a Rabl orientation contain either no ASY1 protein or diffuse ASY1 signals containing brighter, punctate foci always in the same region of the nucleus as the telomeres and weaker DAPI counterstaining (Figure 1A). Differential DAPI staining was frequently observed during leptotene resulting from the polar distribution of pericentromeric heterochromatin in barley (Figure S1C & S1D). Nuclei at the bouquet stage had either the latter pattern of ASY1 described above, or short linear tracts of ASY1 emanating from the nuclear region containing the telomeric cluster (Figure 1D). This indicates that the bouquet forms during leptotene. By the end of leptotene, linear tracts of ASY1 occupy the entire nucleus (Figure S1B). The bouquet is maintained during zygotene (Figure 2E) and dissociates during pachytene (Figure S2A).

These data demonstrate that from the onset of leptotene the majority of centromeres appear to be associated. To test whether or not these aggregates contain homologous pairs of chromosomes, two single-locus BACs of *Brachypodium distachyon* mapping only to the pericentromeric region of the short arm of chromosome 5H of barley (Figure S1C) were hybridised *in situ* together with telomeric and centromeric probes to squashed leptotene meiocytes. Nineteen nuclei with either Rabl (Figure 1C) or bouquet (Figure 1D) configurations have two separate and distant BAC signals in close proximity to different centromere clusters. The bouquet of telomeres is sometimes pinched out from the rest of the nucleus (Figure 1D), and is likely to be the result of mechanical damage during slide preparation. As expected, pachytene nuclei contain seven pairs of centromeres and a single BAC signal lying 0.9 µm (n = 10; SD 0.21) from the 5H centromere, reflecting complete synapsis at this stage (Figures 1E & 1F). This shows that the centromeres of chromosome 5H (and probably other centromeres too) pair non-homologously during leptotene. The apparent size difference between centromeres at early meiosis (Figures S1A) and at pachytene (Figure 1E) can be attributed to the different methods by which these cells were prepared i.e. acrylamide pads for early stages, and squashes for pachytene.

### Progression of synapsis

In order to track at high resolution the progress of synapsis, meiocytes at zygotene of meiosis were embedded in polyacrylamide and two structural proteins, ASY1 and ZYP1, detected using immunolocalisation. The SC-associated protein ASY1 marks unpaired axial elements (AEs) only at this stage, and the transverse filament protein ZYP1 labels synapsed regions (Figure 2A). The ASY1 signal disappears in synapsed regions during zygotene, but reappears during pachytene (Figure 2A & 2E). The disappearance of ASY1 signals during zygotene may result from a temporary masking or modification of its epitope. An alternative explanation is that the protein is removed from the chromosome axes during zygotene and reloaded during pachytene. The nuclei were optically sectioned using confocal laser scanning microscopy (Figure 2A), and the linear tracts of the two proteins traced using Imaris image analysis software (Figure 2B). Each SC was traced from one end to the other; no exchanges of pairing partners were observed, indicating that synapsis was exclusively between homologous chromosomes. The vast majority of ZYP1 sites were at the convergence of two ASY1 strands, and rarely associated with unpaired ASY1 cores (Figure 2C, white box). Each of the seven bivalents per cell were reconstructed (Figures 2B & 2C & Figure S1F), and the absolute lengths of the bivalent and its constituent synapsed and un-synapsed segments recorded. Synapsis starts predominantly at the telomeres clustered in a bouquet (Figure 2E), but other sites of synaptic initiation occur along the length of the bivalents.

A total of nine nuclei were reconstructed, with average percentage synapsis ranging from 38.6 to 98.2. Nuclei with lower percentage synapsis were identified but were not amenable to reconstruction by this method. 57 complete bivalents were isolated and measured from the nine zygotene nuclei, with synapsis ranging from 28.7 to 100%. In three nuclei, 4 partial bivalents were identified and in some instances the partial bivalents terminated in the vicinity of the nucleolus indicating they may represent the nucleolar organising regions found on bivalents 5H and 6H (Figure S1G). Frequent interlocking of chromosomes did not hinder tracking and quantitative analysis as the continuity of the chromosomes in these configurations was not compromised (Figure 2D). Since no interlocks were apparent at pachytene, it is likely that they are resolved by an unknown mechanism during zygotene. Maps of the distribution of synapsed and unsynapsed regions for each bivalent are shown in Figure 2F.

**Figure 3:**
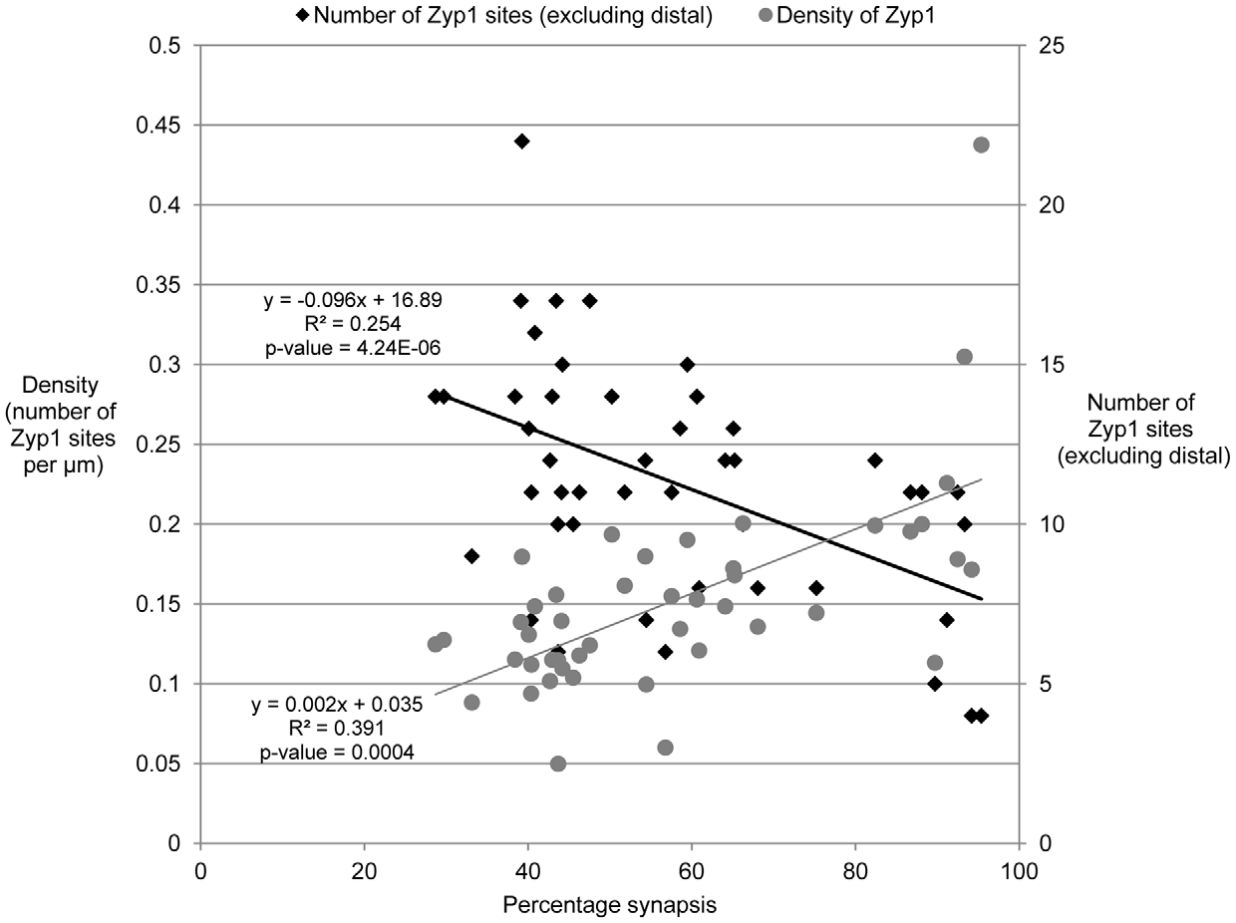
Density of ZYP1 in synapsing bivalents. Plot of the density of ZYP1 fragments in along the synapsing bivalent (grey circles), and the number of ZYP1 sites along the bivalent (excluding distal synapsis) (black diamonds) against percentage synapsis of 45 zygotene bivalents. P-values calculated using a 2 tailed t-test on the slope coefficient in a simple regression.

Forty five of these partially synapsed bivalents (range 29 to 99%) represent the progression of synapsis throughout zygotene. All bivalents have a similar pattern of synapsis, with most of the synapsis in distal regions, but with multiple synaptic sites in interstitial locations too. Figure 3 shows a plot of the number of ZYP1 sites against percentage synapsis for each of the 45 synapsing zygotene bivalents. Despite considerable variation (reflected in the low r^2^ values), the graph shows the trend that bivalents with higher percentage of synapsis have a lower number of sites, which could reflect ongoing synapsis progressively subsuming interstitial ZYP1 sites during zygotene. However, if the density of ZYP1 sites, calculated as the number of ZYP1 sites (excluding the distal synapsis) divided by the combined total length of ASY1 and ZYP1 (excluding the distal synapsis), is plotted against percentage synapsis, there appears to be a reverse trend i.e. bivalents with higher percentage synapsis tend to have denser ZYP1 sites, implying that additional ZYP1 sites may be added as synapsis proceeds (Figure 3). For example, bivalent 2a from Figure 2F is 43% synapsed, has 12 interstitial ZYP1 sites that occupy 117.9µm (total length 156.8µm minus the telomeric synapsed regions (24.2µm+14.7µm)), and has a ZYP1 density of 0.1 foci/µm. Bivalent 7d on the other hand is 87% synapsed, has 11 interstitial ZYP1 sites that occupy a space of only 56.3µm (151.8µm – (56.3µm+79.9µm)), giving a density of 0.2 foci/µm.

### High resolution imaging

**Figure 4:**
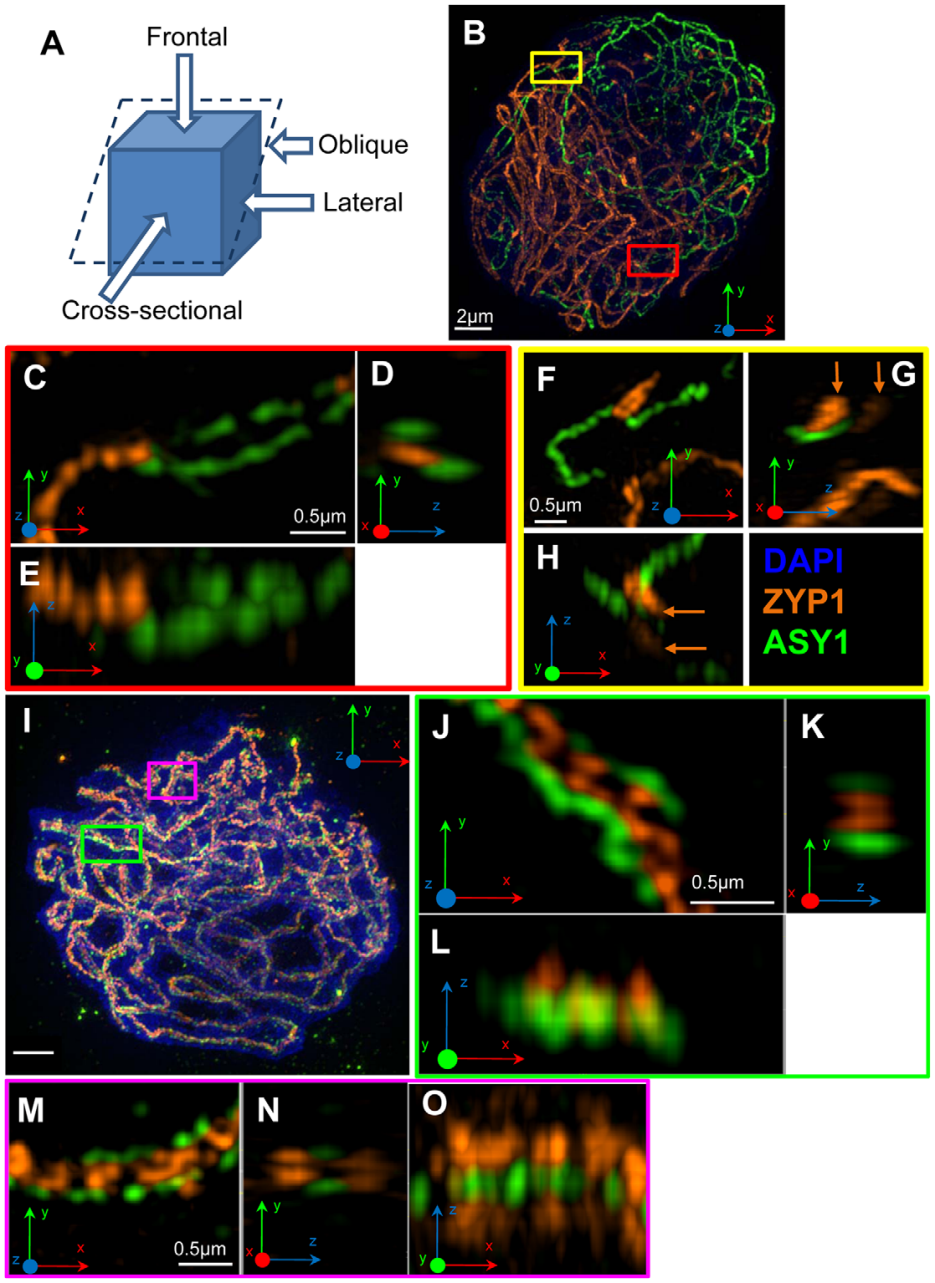
High resolution imaging of barley prophase I nuclei. (A) Diagram illustrating the nomenclature of the various planes of view of the SC, according to Moses (1968). Note that oblique section is defined as any plane of section at an angle to the axis that is not perpendicular. (B) Zygotene nucleus showing ASY1 (green) and ZYP1 (orange) cores, and chromatin (blue). (C–E) Enlargement of the region delimited by the red box in (B) showing the standard SC structure in frontal view (C), cross-sectional view (D) and oblique view (E) where a single ZYP1 structure is visible. (F–H) Enlargement of the region delimited by the yellow box in (B) showing the different SC structure. Frontal view (F), lateral view (G) and oblique view (H) where the two ZYP1 structures are visible (orange arrows) are also shown. (I) Pachytene nucleus showing ASY1 (green) and ZYP1 (orange) cores, and chromatin counterstained with DAPI (blue). (J–L) Enlargement of the synapsed region delimited by the green box in (I) showing the usual SC structure in frontal view (J), cross-sectional view (K) and lateral view (L). (M–O) Enlargement of the synapsed region delimited by the pink box in (I) showing the different SC structure in frontal view (J), cross-sectional view (N) and lateral view (O). All images are maximum projections of nuclei embedded in polyacrylamide and captured by 3D-SIM. The *xyz* angles shown in each image relate to the orientation of the captured image.

**Figure 5:**
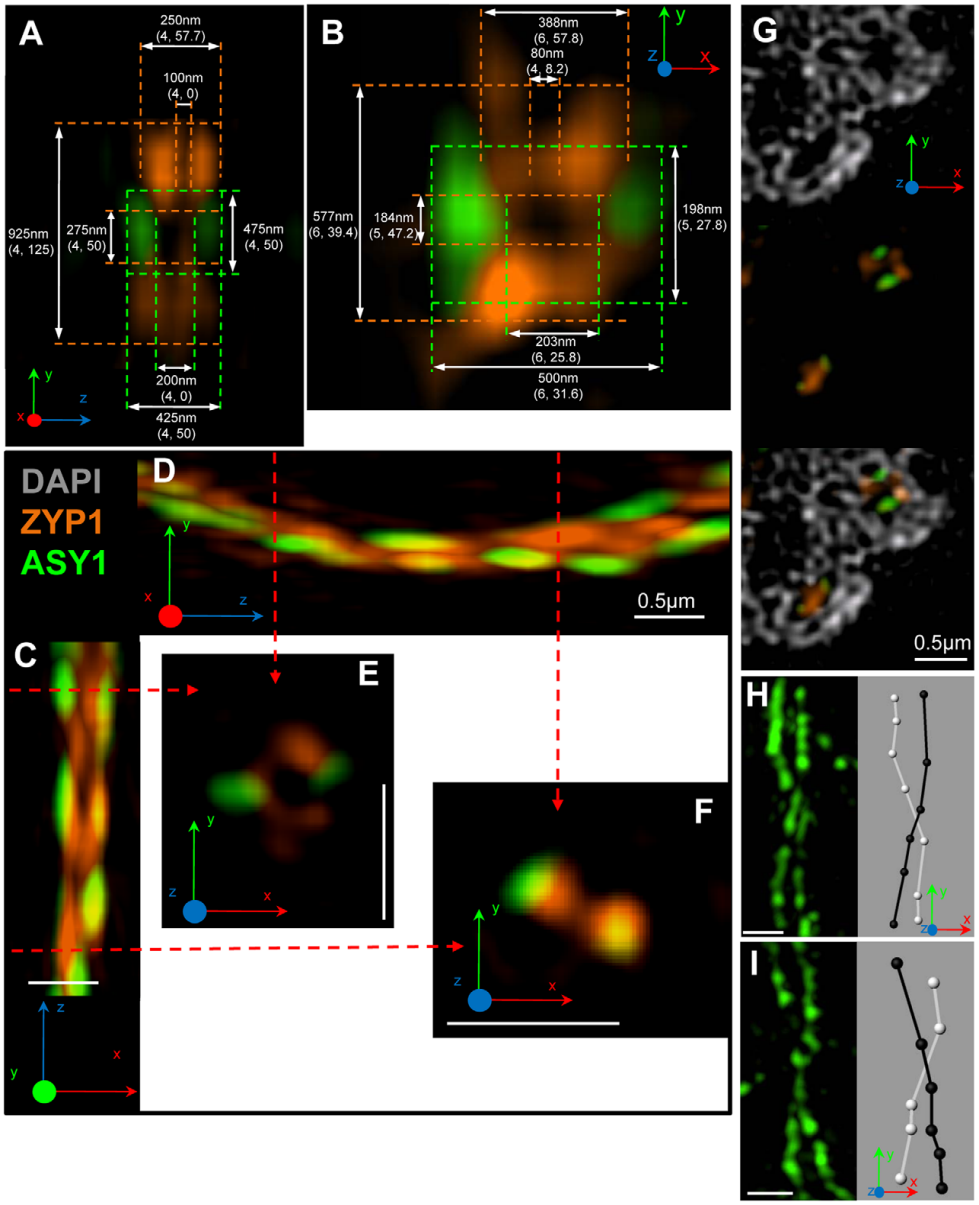
SC structures and features revealed by 3D-SIM. (A & B) Cross-sectional views of the variant SC structure in the *yz* (A) and *xy* planes (B) showing ASY1 (green) and ZYP1 (orange). Average dimensions of the various SC components (the number of measurements taken and the standard deviation shown in parenthesis). (C & D) An SC shown in the *xz* (C) and *yz* (D) planes, with two cross-sectional views (E & F) 2µm apart (red arrows) showing a change in SC conformation. (G) Series of 3 images showing a single *z* section from a limited region of a pachytene nucleus, with DAPI (grey) in the upper pane, two SCs with different conformations in the centre pane and a merged image of the upper two panes. (H & I) Twisting of the LEs during pachytene as revealed by detecting ASY1 protein (green). A left-handed twist (H) and a right-handed twist (I) are shown, together with an interpretive diagram generated in Imaris. All images have been captured by 3D-SIM from pachytene nuclei embedded in polyacrylamide. The *xyz* angles shown in each image relate to the orientation of the captured image.

In order to probe the ultrastructure of the SC beyond the theoretical resolution limit of 200nm of conventional light microscopy, 3D structured illumination microscopy (3D-SIM) was used for the first time to image barley meiocytes. 14 zygotene nuclei were imaged by 3D-SIM. Typical organisation of ASY1 and ZYP1 cores during this stage is shown in Figure 4B. Long tracts of ZYP1 are confined to the region of the nucleus in which telomeres are localised, and shorter ZYP1 fragments are scattered throughout the nucleus. Sites containing ZYP1 are comparable to those imaged by CLSM, insofar as unpaired ASY1 cores converge into tracts of ZYP1 in which ASY1 is no longer detectable (Figure 4C-4E). In these cases, the ASY1 cores converge into a single ZYP1 structure, which is the usual conformation of ZYP1 observed. However, a different SC configuration was also observed in all the zygotene nuclei imaged. In frontal view (see Figure 4A for a visual guide to plane nomenclature), the ZYP1 structures are the same (Figure 4F), and have a mean width of 250nm (n = 4; SD = 58, Figure 5A). However, when the ZYP1 structure is observed from lateral or oblique views (Figures 4G & 4H) the difference between the two structures becomes evident. ZYP1 appears as two parallel tracts separated by a distance of 275nm (n = 4; SD = 50), into which the ASY1 cores converge (Figures 4F & 4H).

**Figure 6:**
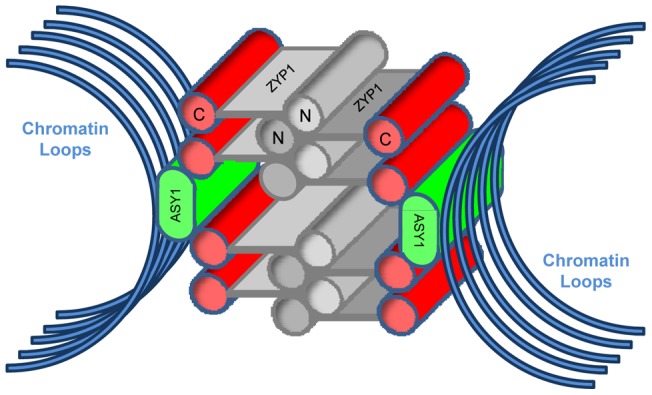
Proposed 3D model of the variant SC structure. Proposed 3D model of the variant SC structure based upon published information about ZYP1 and ASY1 proteins and the 3D-SIM images described in this study. Chromatin loops (blue) are shown to attach in the vicinity of the ASY1 protein (green). The C-terminus of the ZYP1 protein (red) is the epitope for its antibody. The precise location of the LEs is currently unknown, so they have been excluded from the diagram.

A total of 19 pachytene nuclei were imaged using 3D-SIM (Figure 4I). Figure 4J shows a typical frontal view of a classical tripartite structure with an average width of 425nm (n = 4; SD = 50), comprising two ASY1 cores separated by a gap of 200nm (n = 4; SD = 0) enclosing a ZYP1 core. In the lateral view and cross-sectional view, only one ZYP1 core is evident comprising two substructures separated by 100nm (n = 4; SD = 0) (Figures 4K & 4L). The ZYP1 antibody was raised against the C-terminus of the protein which is known to interact with the LE. The two substructures observed likely represent either end of the two ZYP1 proteins that form the central element (CE) (Figure 6). In addition to the standard tripartite SC structure expected from previous EM studies, 3D-SIM has unveiled an SC structure that is strikingly different. Clearly, the SC is the expected tripartite sandwich of ZYP1 flanked by two ASY1 cores in the frontal view only (Figure 4M). In other views (lateral and cross-sectional), ZYP1 appears as two distinct elements, each of which comprises two substructures (Figures 4N & 4O). The ASY1 cores when viewed from the lateral view (Figure 4O) appear to sit in the centre of the two ZYP1 cores. Detail of a synapsed region in three different planes is shown in Figures 4M-4O, and the various dimensions of the constituent parts in cross-sectional view are shown in Figures 5A (*yz* plane) and 5B (*xy* plane). The different SC structure is unlikely to be a technical artefact due to rendering with Imaris software, as it is discernible in multiple planes (*xy* and *yz*) and in consecutive sections in the *z* plane (Figure S2H).

The significance of the two SC structures observed during zygotene and pachytene is not known. Neither SC structure is confined to a particular region or synaptic event (both structures were observed at pairing forks), and both are seen in close proximity in the same SC (Figure 5C-5F) and in different SCs within the same *z* section in the *xy* plane (Figure 5G). The relative frequencies of each SC structure could not be ascertained due to the lack of continuity of the ASY1 cores and the difficulties in viewing the SC in cross section over long lengths of axis.

Left-handed and right-handed twists of the two parallel ASY1 cores were observed (Figures 5H & 5I), together with long lengths of parallel lateral elements (LEs) containing no twists (Figure S2G). The lack of continuity of ASY1 signal at pachytene precluded an estimation of the relative frequencies of right-handed and left-handed twists.

3D-SIM has unveiled two different SC structures which are common during zygotene and pachytene, and one of them is strikingly different from the standard tripartite structure usually reported.

## Discussion

### Behaviour of chromosomes at early meiosis

The behaviour of chromosomes at early meiosis was investigated by tracking centromere and telomere domains by FISH. Nuclei entering meiosis have a bipolar Rabl orientation of these domains, which is consistent with previous observations of somatic interphase cells in barley [37]. The telomeres aggregate during the leptotene stage to form a classic bouquet, the timing of which is similar to maize [38], but later than wheat and rye which form bouquets at the onset of meiosis [25], [39], [40]. The nature of the telomeric associations in nuclei with a bouquet was not ascertained, but the close approximation of the number to the basic chromosome number of seven of this species could indicate that the ends of homologous bivalents associate preferentially.

During leptotene, all but one of the nuclei analysed had fewer than 14 centromere signals, indicating that centromere association is a regular feature of meiosis at this stage in barley. Centromeres associate at early meiosis in other members of the Poaceae, such as *Aegilops squarrosa* and *Triticum monococcum* [25], and allohexaploid *T. aestivum* which forms seven groups of homoeologous centromeres [41]. The reduction in mean number of centromere aggregates to 7.46 at the bouquet stage suggests that the associations are homologous. However, at least with respect to chromosome 5H, this is not the case as a pair of pericentromeric, single-locus tags were not associated together in any of the centromeric clusters observed. Nuclei containing 5–11 centromere signals were recorded, implying that the associations were not simply a pair-wise coupling of centromeres. Although this is the first time that non-homologous association of centromeres has been shown in plants at leptotene using FISH, the same phenomenon has been described before in budding yeast [42]. In this organism, it was shown that Rec8 and Zip1 (orthologous to ZYP1 of plants) proteins were required for centromere coupling and localised to centromeres during early meiosis [42], [43]. No ZYP1 protein was detected immunologically in centromeres at leptotene in barley (Figure S1H), although this does not rule out the possibility that low levels of this protein maintain centromere associations at this stage.

The coupling of non-homologous centromeres during leptotene at a time when homologues are preparing for synapsis appears counterintuitive. However, it may be part of a mechanism to inhibit reciprocal recombination in centromeric regions [44] which is known to make chromosomes vulnerable to non-disjunction at anaphase I in human, *Drosophila* females and budding yeast [45], [46]. The lack of a homologue in these regions would force any incipient recombination event along a non-crossover pathway.

In order to couple the behaviour of telomeres to the assembly of meiotic chromosomes, FISH was used in conjunction with the immunolocalisation of SC-associated protein ASY1 which has proven to be a reliable marker for AEs and LEs in *Arabidopsis* [32], rye [19] and barley [20]. ASY1 protein appears as an amorphous cloud of signals at early leptotene in the same region of the nucleus as the polarised telomeres. This polar localisation has only been described in barley to date and has not been observed in *Arabidopsis* [32], rye [18], [19], maize [47], wheat [16], nor in rice with respect to the orthologous protein PAIR2 [48]. This region of the nucleus also has a lower density of DAPI staining, which usually indicates the presence of predominantly euchromatin. Differential DAPI staining appears, therefore, to reveal a polarised distribution of euchromatin and heterochromatin during early meiosis in barley. This has been noted before in somatic interphase nuclei of barley, and was also attributed to the polar distribution of heterochromatic DNA [37]. It is interesting to speculate that the co-localisation of euchromatin and ASY1 at this stage may be functionally related, and that this polarity may be connected with the preferential synapsis of telomeres in this species, and with the distal localisation of chiasmata. There was no discernible correlation between the behaviour of telomeres and the polymerisation of ASY1. The retention of the telomere cluster during zygotene and its dispersal during pachytene are similar to maize [38], humans [49] and budding yeast [50].

### Progression of synapsis

Immunolocalisation of two SC proteins has been used for the first time in barley to describe quantitatively the progression of synapsis. The data show that although synapsis is preferentially initiated at, and driven from, the telomeres, multiple sites of synaptic initiation occur along the length of zygotene bivalents. This pattern of SC formation is common to other plant species, such as rye [51], [52], lily [53], [54] and *Tradescantia* [55], [56]. The loss of ASY1 from the synapsed axes has been previously reported in maize [47], and in rice the intensity of the PAIR2 signal is significantly diminished in synapsed axes [48].

The number of discrete, interstitial ZYP1 sites in the 45 synapsing bivalents analysed ranged from four to 23, and appeared not to correlate with the length of the bivalent. The downward trend in the number of sites per bivalent as synapsis proceeds could be attributable to the coalescing of sites. If this were true, the density of interstitial sites would be about the same in bivalents with different extents of synapsis. The density of ZYP1 sites is actually higher in bivalents with more advanced synapsis, indicating that ZYP1 sites are being added as synapsis progresses.

The precise function of the ZYP1 sites in barley is not known, but studies of the orthologous protein (Zip1) in budding yeast have shown that they are connected with synapsis initiation complexes (SICs) [42], [57], [58], [59]. SICs of yeast are located at sites of axial associations where two homologues become closely juxtaposed [57], [60], and contain Zip2 which is dependent upon double-strand break formation by Spo11 [57], [58]. All sites containing ZYP1 identified in this study formed between pairs of homologous chromosomes, which supports this hypothesis. If SICs in barley represent potential sites of recombination, it must be assumed that the majority are resolved by a non-crossover pathway, since chiasmata are distally localised in this species. A higher number of SIC sites compared to the number of mature recombination events has been observed in many other plant species including *Arabidopsis*, rye, lily and *Tradescantia* [52], [54], [55], [61]. SICs in budding yeast are the sites of SC elongation, but do not guarantee that elongation will occur [57]. The large number of small ZYP1 sites in largely unsynapsed bivalents of barley compared with the relatively few longer stretches of ZYP1 in later bivalents, coupled with the supposed addition of ZYP1 sites as synapsis proceeds, suggests that only a subset of SICs elongate in barley too.

### A new perspective on SC structure

3D-SIM enables the imaging of structures less than 100nm in the *xy* plane and less than 250nm in the *z* plane [62], [63], [64], providing an alternative method to electron microscopy for dissecting the substructure of SCs. 3D-SIM been used only once before to study meiosis – to resolve two AE proteins of maize [47]. This present study is the first to use super-resolution light microscopy to probe the substructure of both CE and AE/LE components of the SC.

Anti-ASY1 antibody faithfully highlights un-synapsed AEs during zygotene, but does not detect its protein once it is complexed with ZYP1 at SC initiation sites. The same observation has been made at high resolution in maize [47]. ASY1 protein is detectable later at pachytene, although not as a continuous signal along the entire length of the SC. Right- and left-handed twists of LEs are observed in fully formed SCs, but do not coil as those observed in maize [47]. The twists observed in barley are more reminiscent of those described in EM studies of the SC in plants such as rye [51], [52].

3D-SIM reveals the SC in a frontal view as a tripartite structure comprising a central, linear tract of ZYP1 protein flanked by two linear rods of ASY1 protein. This ultrastructure is consistent with that imaged by 3D-SIM in maize [47] and CLSM in rye [18], and bears close similarity to the highly conserved tripartite structure in EM studies of numerous organisms [29], [31], [65]. However, if the SC is observed from a lateral or cross-sectional view, two different structures are revealed by the ZYP1 antibody at both zygotene and pachytene. One SC structure conforms to the classical model of the SC. The other comprises two ZYP1 structures which flank and make contact with the two ASY1 elements (see the model in Figure 6). The variant SC structure identified in zygotene nuclei is more difficult to interpret due to the lack of ASY1 signal. It is feasible that the secondary ZYP1 structure may be an aggregation of ZYP1 protein or a form of polycomplex, although the alignment of the synapsing ASY1 cores and the dimensions of the ZYP1 structure strongly suggest it is the same variant SC as that seen at pachytene.

In the different SC structure identified, the two ZYP1 structures lie above and below the transverse plane of the SC, thereby increasing substantially its overall dimensions. Only the 100nm space between the two ZYP1 substructures is consistent with the classical width of the central region of the SC. Since the ZYP1 antibody was raised against the C-terminus of the protein which is known to interact with LEs, the close apposition of the two proteins is not unexpected. The overall average dimensions of the two forms of the SC in cross-sectional view depend upon whether the image is captured in the *xy* plane or *yz* plane. Due to the lower resolution in the latter, the measurements made in the *xy* plane are more reliable. In either plane, the overall dimensions of the structure is much larger than previously estimated by EM raising the possibility that the SC is much larger [30], [66]. Although Gillies [31] reported uniformity in SC dimensions in EM studies of 31 plant species, variant SC structures have been observed in plants, such as polycomplexes in *Allium cepa* [67] and bipartite LEs and central elements in lily [68]. Considerable variation in SC conformation has also been recorded in a wide range of organisms from many genera [30], [65], [69]. The variant SC structure observed in this study compared to those obtained by EM could be the result of the relatively short fixation time and subsequent incubations in aqueous solutions of the immunocytological method employed. In addition, the detection of SC proteins using primary and secondary antibodies may magnify the dimensions of their targets. The results obtained in this study suggest that the SC of barley has a fluid structure, which is in keeping with previous reports, such as those offering rapid desynapsis and resynapsis as a possible means of interlock resolution [30].

### Concluding remarks

High resolution 3D reconstruction of meiotic nuclei has shown that polarised loading of ASY1 protein, clustering of telomeres, preferential synapsis from the telomeres, and non-homologous association of centromeres are regular features of early meiosis in barley. The question remains as to how these events are functionally related, and to what extent they may influence the distal localisation of chiasmata. It is tempting to speculate that one or more of these processes may predispose in a temporal sense the distal regions of chromosomes to crossover events. If this were to be the case, changing the early associations of chromosomes or the patterns of synapsis may be profitable interventions in terms of manipulating recombination in this species. It is not at present known the significance of the different forms of the SC, and what specific roles they may play in the recombination process.

## Materials and Methods

### Plant material

Barley (*Hordeum vulgare* cv. Morex (2n = 2x = 14)) was grown to maturity under 16h days with 60 µmol/m^2^/sec illumination at a constant 20°C in standard greenhouse conditions.

### Preparation of mitotic and meiotic chromosome squashes and FISH

Barley seeds were germinated and treated as described by Cuadrado *et al.* [70] and mitotic chromosomes were prepared according to Jenkins *et al.* [71]. Meiotic chromosomes were prepared as described by Idziak *et al.* [72]. A 2.3kb subclone of 25S rDNA from *A. thaliana* [73] was labelled by PCR with biotin-16-dUTP (Roche) as described by Mikhailova *et al.* [18]. Centromeric [74] and telomeric [75] sequences were labelled with tetramethyl-rhodamine-5-dUTP (Roche) by PCR [20]. Single-locus, centromeric BACs DH053N18 and DH096P22 derived from *Brachypodium distachyon* [76] were labelled with digoxigenin-11-dUTP (Roche) using nick translation (Roche) as described in Jenkins *et al.* [71]. FISH was performed largely as described in Phillips *et al.* [20] with the following modifications. Mitotic chromosomes were denatured for 6.5 min at 75°C, and stringent washes were carried out in 0.1× SSC at 42°C for 2×10 min. Meiotic chromosomes were denatured for 5 min at 75°C and a stringent wash was carried out in 20% formamide in 2× SSC at 37°C for 10 min. Digoxigenin and biotin were detected by fluorescein anti-digoxigenin antibody (1 ∶20, Roche) and Cy5-streptavidin (1∶250, Invitrogen), respectively.

### Acrylamide embedding of barley meiocytes

Barley meiocytes were embedded in acrylamide in order to preserve their three dimensional architecture. The method of Bass *et al.* [38] was adopted, with the following modifications. Anthers at the desired stage of meiosis were harvested into Buffer A and fixed for 10 min in freshly prepared 2% paraformaldehyde in Buffer A. Anthers were washed twice in Buffer A and macerated using a brass rod in Buffer A. The meiocyte suspension was then embedded in acrylamide as described by Bass *et al.* [38].

### Sequential immunolocalisation and FISH in polyacrylamide pads

The polyacrylamide pads were processed as previously described in Phillips *et al.* [20]. Briefly, pads were incubated in blocking buffer containing anti-ASY1 antibody raised in rabbit [32] and in some instances anti-ZYP1 raised in rat [33] both diluted 1∶250 for 36h at 4°C. Pads were washed 3×30 min in PBS+0.1% Tween 20+1mM EDTA pH 8 at room temperature followed by fixation in freshly prepared 2% paraformaldehyde in Buffer A for 30 min at room temperature and 3×30min washes in PBS+0.1% Tween 20+ 1mM EDTA pH 8 at room temperature. FISH with telomere, centromere and 25S rDNA probes was performed according to Mikhailova *et al.* [18] with the following modifications. Chromosomes were denatured for 8 min at 75°C, followed by two stringent washes in 0.1× SSC at 37°C for 30 min each. Pads were incubated overnight at 4°C with, where appropriate, Alexa Fluor 488 anti-rabbit antibody (Molecular Probes), Alexa Fluor 546 or Alexa Fluor 594 anti-rat antibodies all diluted 1∶250 in blocking buffer, and fluorescein anti-digoxigenin antibody diluted 1∶20 in blocking buffer. The pads were washed 3×30 min in PBS+0.1% Tween 20+1mM EDTA pH 8 at room temperature followed by a 30 min wash in PBS before being mounted in mounting medium (200mM Tris-HCl pH 8, 2.5% DABCO (1,4-diazobicyclo(2,2,2)octane), 80% glycerol and 1µg/ml DAPI.

### Image acquisition and analysis

Nuclei were optically sectioned using either a Leica DM6000B wide-field fluorescence microscope equipped with a Leica DFC350 FX R2 camera controlled by Leica LAS-AF software, or a Leica TCS SP5II confocal laser scanning microscope (CLSM) controlled by Leica LAS-AF software. Z-stacks were deconvolved using AutoQuant X2 (Media Cybernetics) and analysed using Imaris 7.3 (Bitplane). Imaris allows the Z-stacks to be rendered in 3D and in this space surfaces were manually added to trace each of the bivalents. Three-dimensional structured illumination microscopy (3D-SIM) was performed on an OMX version 2 microscope system (Delta Vision; Applied Precision). Raw 3D-SIM images were processed and reconstructed with SoftWorx version 4.5.0 and subsequently each channel aligned using SoftWorx alignment tool (Applied Precision).

## Supporting Information

Figure S1 (**A**) **Leptotene nucleus containing a bouquet of telomeres** (**red**) **and associated centromeres** (**red**)**.** (B) Leptotene nucleus containing continuous ASY1 cores (green). (C) Condensed mitotic chromosomes at metaphase showing FISH of single-locus BAC DH053N18 (green; green arrows) landing to the pericentromeric region of the short arm of chromosome 5H, and 25S rDNA loci (yellow; yellow arrows). (D) Leptotene nucleus containing polarised ASY1 signals (green) and a tight bouquet of telomeres (red). (E) Same nucleus shown in (D) showing the DAPI channel only, clear polarisation of DAPI evident, with the lightly staining chromatin co-localising with the ASY1. (F) Zygotene nucleus processed using Imaris showing the reconstruction of all synapsing bivalents, with generated surfaces for ASY1 (green), ZYP1 (red) and a yellow sphere delimiting the approximate size and position of the nucleolus. (G) One short and one long partial bivalents extracted from the reconstruction in (F). (H) Leptotene nucleus containing rendered spheres delimiting the position of the centromeres (green) and ZYP1 (red). All images except (C) are deconvolved maximum projections of meiotic nuclei embedded in polyacrylamide and captured using CLSM (A, D-H) or 3D-SIM (B). (C) is a deconvolved maximum projection imaged by wide-field fluorescence microscopy. All chromatin is counterstained with DAPI (blue/grey).
(TIF)

Figure S2 **(A) Image of a pachytene nucleus containing ASY1 (green), ZYP1 (orange) and 14 telomeres delimited by red spheres.** ***yz*** **section (B) and** ***xz*** **section taken from the pachytene nucleus shown in (A).** An enlarged frontal view (D) and lateral views (E & F) of the SC structures from the region delimited by the white box in (A). (G) Two LEs highlighted by the ASY1 antibody (green) running in parallel for 11µm, together with an interpretive diagram of the LEs generated by Imaris showing absence of twisting. (H) Consecutive images through the *z* plane of the SC structure shown in (D). All images have been captured by 3D-SIM from pachytene nuclei embedded in polyacrylamide. The *xyz* angles shown in each image relate to the orientation of the captured image.
(TIF)
