# Supplementary figures and images for: High Resolution Analysis of Meiotic Chromosome Structure and Behaviour in Barley (Hordeum vulgare L.)
Source: PLoS One. 2012 Jun 25;7(6):e39539. doi: 10.1371/journal.pone.0039539 (PMC3382580; doi:10.1371/journal.pone.0039539)

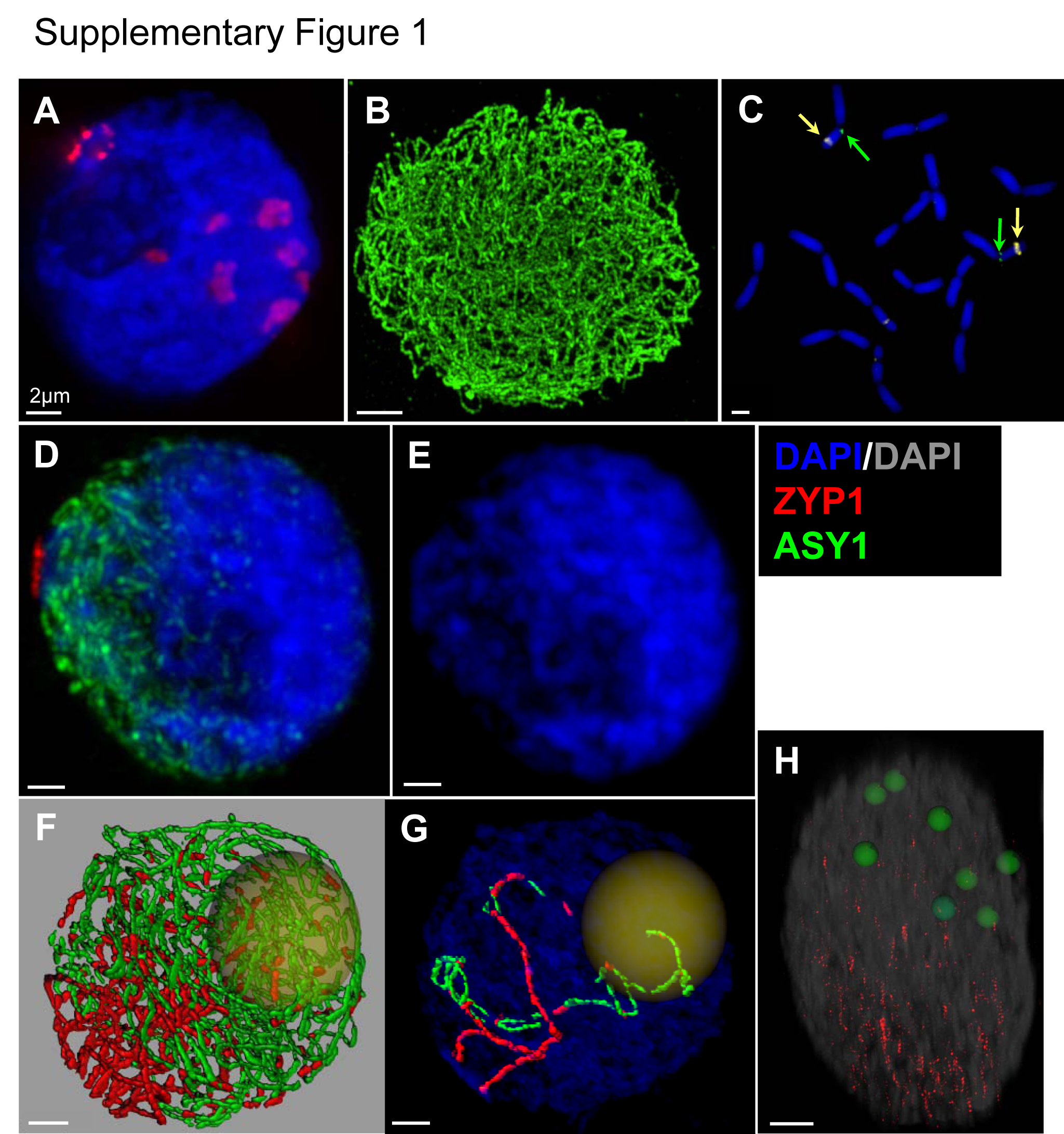

Supplement: Figure S1 — (A) Leptotene nucleus containing a bouquet of telomeres (red) and associated centromeres (red). (B) Leptotene nucleus containing continuous ASY1 cores (green). (C) Condensed mitotic chromosomes at metaphase showing FISH of single-locus BAC DH053N18 (green; green arrows) landing to the pericentromeric region of the short arm of chromosome 5H, and 25S rDNA loci (yellow; yellow arrows). (D) Leptotene nucleus containing polarised ASY1 signals (green) and a tight bouquet of telomeres (red). (E) Same nucleus shown in (D) showing the DAPI channel only, clear polarisation of DAPI evident, with the lightly staining chromatin co-localising with the ASY1. (F) Zygotene nucleus processed using Imaris showing the reconstruction of all synapsing bivalents, with generated surfaces for ASY1 (green), ZYP1 (red) and a yellow sphere delimiting the approximate size and position of the nucleolus. (G) One short and one long partial bivalents extracted from the reconstruction in (F). (H) Leptotene nucleus containing rendered spheres delimiting the position of the centromeres (green) and ZYP1 (red). All images except (C) are deconvolved maximum projections of meiotic nuclei embedded in polyacrylamide and captured using CLSM (A, D-H) or 3D-SIM (B). (C) is a deconvolved maximum projection imaged by wide-field fluorescence microscopy. All chromatin is counterstained with DAPI (blue/grey). (TIF) [file pone.0039539.s001.tif]

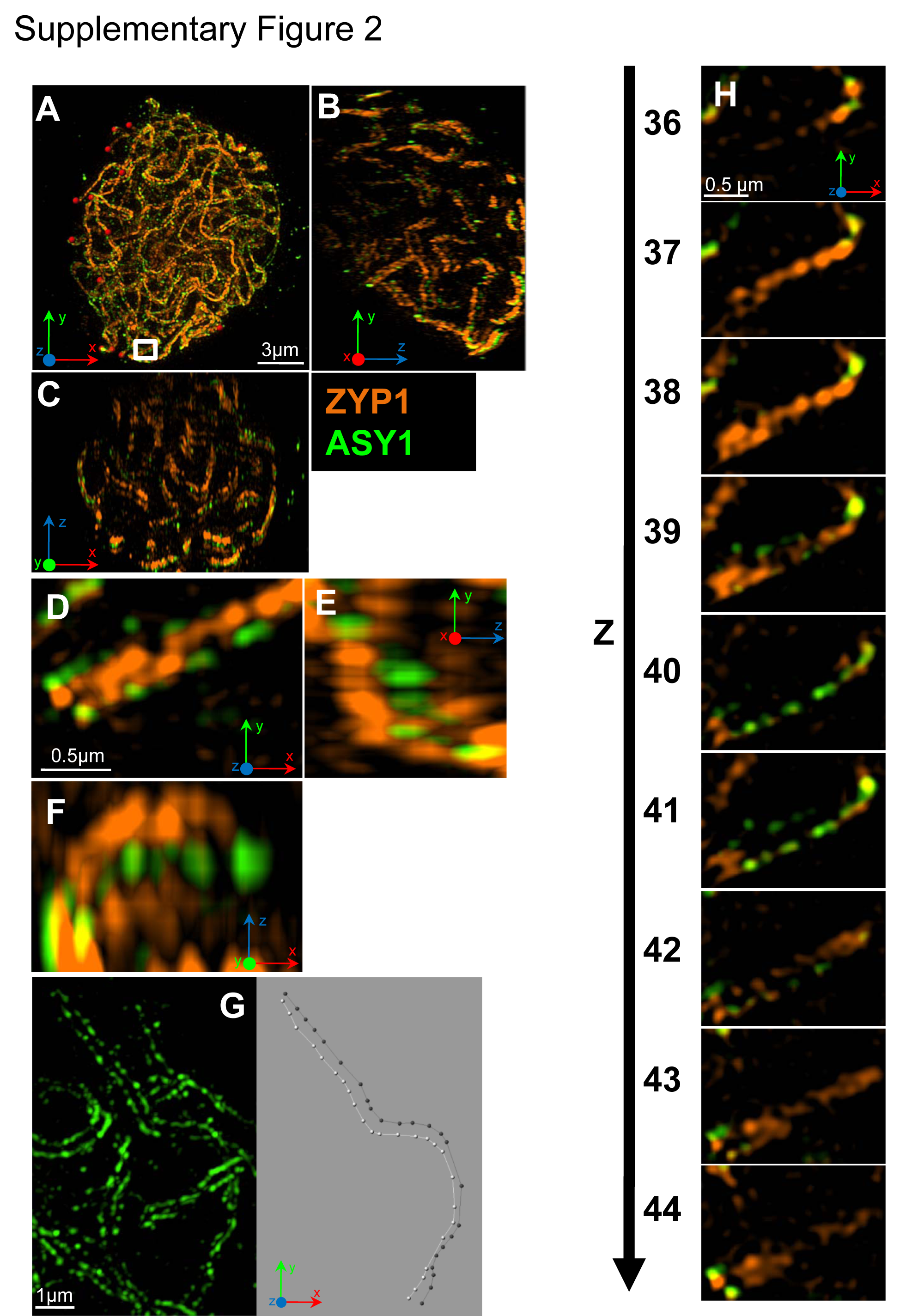

Supplement: Figure S2 — (A) Image of a pachytene nucleus containing ASY1 (green), ZYP1 (orange) and 14 telomeres delimited by red spheres. yz section (B) and xz section taken from the pachytene nucleus shown in (A). An enlarged frontal view (D) and lateral views (E & F) of the SC structures from the region delimited by the white box in (A). (G) Two LEs highlighted by the ASY1 antibody (green) running in parallel for 11µm, together with an interpretive diagram of the LEs generated by Imaris showing absence of twisting. (H) Consecutive images through the z plane of the SC structure shown in (D). All images have been captured by 3D-SIM from pachytene nuclei embedded in polyacrylamide. The xyz angles shown in each image relate to the orientation of the captured image. (TIF) [file pone.0039539.s002.tif]
